# Clustering in Mixtures of SALR Particles and Hard Spheres with Cross Attraction

**DOI:** 10.1021/acs.jpcb.1c09758

**Published:** 2022-02-28

**Authors:** Gianmarco Munaò, Santi Prestipino, Jean-Marc Bomont, Dino Costa

**Affiliations:** † Dipartimento di Scienze Matematiche e Informatiche, Scienze Fisiche e Scienze della Terra, Università degli Studi di Messina, viale F. Stagno d’Alcontres 31, 98166 Messina, Italy; ‡ Université de Lorraine, LCP-A2MC, UR 3469, 1 Blvd. François Arago, Metz F-57078, France

## Abstract

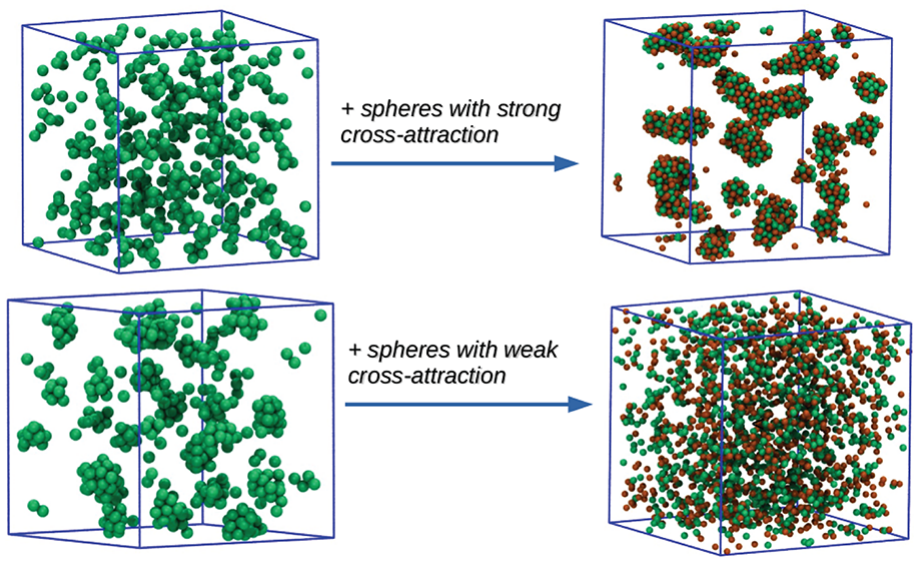

Self-assembling complex fluids are often modeled as particles with effective competing isotropic interactions, combining a short-range attraction (SA) followed by a longer-range repulsion (LR). For moderately low temperatures and densities, SALR particles form clusters in equilibrium, at least provided that the potential parameters are appropriate. Here we inquire into the possibility that cluster formation in SALR fluids might be pushed by a foreign species even under thermodynamic conditions that would not allow for clusterization of the pure system. To this aim, we study by Monte Carlo simulations a mixture of hard-sphere two-Yukawa particles and hard spheres, with a cross interaction modeled by a square-well attraction, and we investigate the conditions of clustering in terms of strength of attraction and relative concentration of the two species. We find that clusters can occur in the mixture for the same temperature and density where the pure SALR fluid is almost structureless. In particular, we single out a cross attraction such that clusters are formed with a SALR concentration as low as 5%. We also find a situation where nearly pure droplets of hard spheres are held together by a shell of SALR particles. Conversely, we show that clustering can be undermined in the mixture under conditions for which this process takes place in the parent SALR fluid. Using a simple criterion, based on the second virial coefficients of the attractive part of interaction potentials (the so-called “reference attractive fluids”), we are able to predict accurately whether clustering is favored (or hindered) in the mixture, as compared to the pure SALR fluid.

## Introduction

Nowadays, it is well recognized that the phase behavior of a complex fluid can be significantly rich and intriguing, owing to its ability to self-assemble into aggregates exhibiting modulated patterns at the mesoscale.^1−7^ In this context, a large variety of colloidal dispersions, as well as block copolymer melts and solutions of amphiphilic molecules, can be described by effective pair interactions featuring a short-range attraction (SA) followed, at larger distances, by a long-range repulsion (LR) .^8−14^ While the SA component, originating from van der Waals and/or depletion forces, favors particle aggregation, the LR component arises from electrostatic forces and imposes a penalty on the growth of a dense macroscopic phase. The distinguishing feature of SALR fluids is their attitude to self-assemble into equilibrium structures of various morphologies^15−23^ characterized by spatial inhomogeneities whose typical length is larger than the particle size, but definitely not macroscopic. The size and shape of these patterns strongly depend on the detailed force parameters, as well as on the values of the thermodynamic variables (i.e., the temperature *T* and the density ρ). Generally speaking, the knowledge of this dependence can be exploited to steer the process of self-assembling toward the formation of the desired structure. For example, for moderately low values of *T*, one expects to see clusters in the dilute fluid, giving way to a percolated fluid at higher densities.^18,19,24,25^ Using density-functional theory, it has been shown that, for sufficiently low temperatures, SALR particles give rise to periodic microphases exhibiting (in increasing order of density) clusters, cylinders, lamellae, inverted cylinders, and inverted clusters, just to name the simplest structures.^26−29^ The equilibrium phase diagram of a cluster-forming fluid has been also reconstructed by a thermodynamic model and assessed by computer simulation.^30^ The broad interest in the physics of SALR fluids is witnessed by four recent reviews on this subject.^31−34^

If we restrict to low-density conditions, such that the self-organized aggregates would essentially be clusters, the crucial problem is clearly how to establish the occurrence of clusters in the simulation sample. From a structural point of view, the characteristic condition for the development of microphases would be a maximum in the static structure factor *S*(*k*) at a wavenumber *k*_0_ ≠ 0, well below the location of the main diffraction peak;^34−36^ by contrast, a diverging *S*(0) invariably implicates liquid–vapor separation. In fact, in a hard-sphere two-Yukawa (HSTY) fluid—a prototypical instance of an SALR fluid—the existence of a low-*k* peak in *S*(*k*) is not by itself sufficient to identify a cluster phase;^18,19,35,36^ rather, this peak is indicative of the development of some kind of modulation (intermediate-range order), in the fluid. As a matter of fact, a low-*k* peak can occur under four different structural conditions, i.e., a monomer-dominated fluid, a cluster fluid, a random percolated fluid, and a cluster percolated fluid, as can be discriminated from the shape of the cluster-size distribution (CSD).^18,19^ In particular, a monotonically decreasing CSD represents a fluid of nonbonded particles, whereas the presence of a maximum in the CSD unambiguously identifies a cluster fluid. When more than 50% of the particles are collected in the same network, the system undergoes percolation. If the CSD exhibits a single peak at a size comparable with the system size, then we speak of a random percolated fluid. On the other hand, if in addition to this peak the CSD also shows the maximum typical of a cluster fluid, then we have a cluster percolated fluid. Interestingly, when the height of the low-*k* peak exceeds a value ≈3, then the system is either a cluster fluid or a cluster percolated fluid.^18,19^ This empirical rule-of-thumb, reminiscent of the Hansen–Verlet criterion for freezing of simple fluids,^37^ is a useful means to locate the onset of a cluster phase. Other criteria, based on structural indicators, have been proposed to identify the onset of clustering in SALR fluids, either in terms of specific features of the low-*k* peak^38,39^ or in terms of the properties of real-space correlations.^40−42^ As for thermal properties, a maximum is generally expected in the constant-volume specific heat at the crossover between the monomer- and the cluster-dominated fluid, since here a randomly selected particle can equally be found as isolated or belonging to a cluster of nearly optimal size.^43^ This leads to strong energy fluctuations and thus to a specific-heat peak, which then provides a practical, easily accessible tool to discriminate between different aggregation regimes.^43^

So far, our discussion has been limited to one-component SALR fluids, though it is clear that the self-assembling scenario of a binary mixture is potentially far richer. In this regard, one way to induce a spatially modulated pattern in a mixture with attractive and repulsive short-range forces would be to employ a suitable molecular structure for one of the species; see, e.g., ref (44). Recently, we have followed this route in a number of papers^45−51^ where we have reported on a variety of self-assembling structures in a mixture made up of amphiphilic dimers and hard spheres. A second possibility would be to adopt a fully coarse-grained view to the problem, by considering a mixture of monatomic species, but then we must leverage on the shape of interactions to produce stable microphases. Examples of such mixtures are found in refs (52 and 53). In these studies, the particle core of both species is harshly repulsive, self-interactions are of SALR type, and the cross interaction is repulsive in the core and attractive at short distances. The force parameters are so tuned that, for sufficiently low *T* and ρ, a one-component system of SALR particles forms clusters. In another study, the self-interaction of one species was purely attractive outside the core.^54^ Then, if the cross interaction is weak, this species undergoes liquid–vapor separation at low *T*. For a strong cross attraction, the resulting clusters are composed of the two species in more or less equal proportions.

In the present paper, we study a mixture of two monatomic species: type-1 particles interact via a HSTY potential, whereas type-2 particles are simple hard spheres (HS). Outside the core, the cross interaction is a square-well (SW) potential of depth ε_12_. The proposed model would represent a generic colloidal mixture where species 1 is a cluster-former, whereas particles of species 2 are relatively blind to each other, even though—due to its affinity to species 1—they will actively participate in the formation of aggregates. Moreover, our system can describe the large class of soft materials—as for instance solutions of biomolecules—in which solutes interact through screened-Coulomb potentials with other species. Our mixture has similarities with (and also differences from) the model investigated in ref (54). For instance, the type-2 interaction in the latter model reduces to hard-core interaction in the limit of vanishing attraction. On the other hand, the cross attraction is different, being a SW here and a Yukawa attraction in ref (54). Our choice possibly allows one to more effectively discriminate the role played by the cross interaction in the overall organization of the mixture. Finally, in ref (54), the mechanism of clustering is mainly investigated through a thermodynamic model, whereas our approach is exclusively simulation-based.

Once established the above-mentioned setup, two questions will be addressed in the following: first, the role played by HS particles in the formation of clusters of HSTY particles, as a function of ε_12_ and relative concentration. Second, the conditions under which the HSTY particles are able to induce droplets of HS particles. In particular, it would be interesting to know whether the HSTY particles can form a coating shell around the HS particles, as it occurs for amphiphilic dimers in mixtures with a small fraction of hard spheres.^47,49−51^

The rest of the paper is organized as follows. In Section II we describe the model and the simulation method to perform the investigation. Sections III–V contain the core of our study: first, we focus on the pure HSTY fluid (Section III), establishing the temperature below which a cluster phase is stable for a specific low value of the density. This analysis is preliminary to the study of a HSTY–HS mixture, for which we examine the effect of strengthening the cross interaction (Section IV) or changing the relative concentration (Section V), until we clarify the exact terms of the interplay between the two species with regard to clustering. Some concluding remarks follow in Section VI.

## II Model and Methods

**Figure 1:**
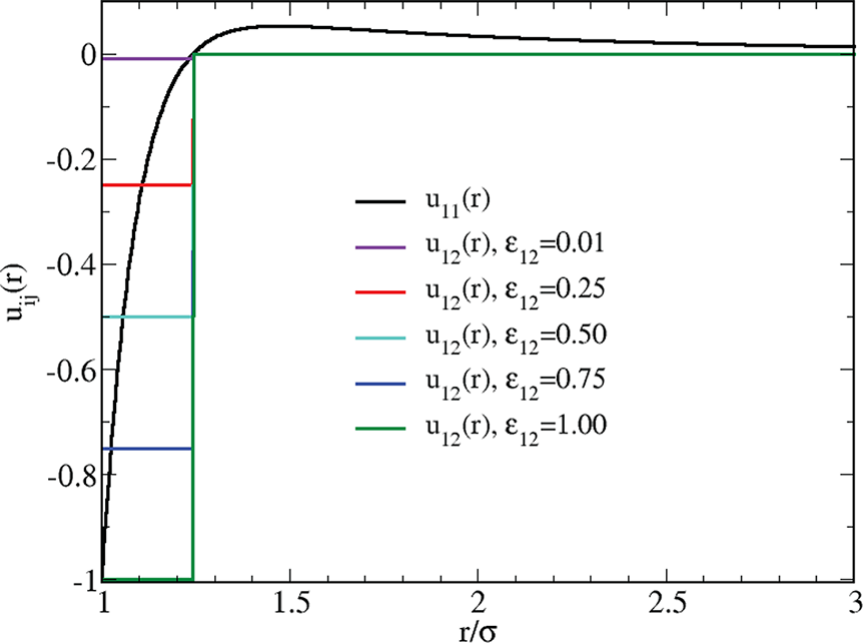
Interatomic potentials investigated in this work: the HSTY potential *u*_11_(*r*) (eq 1, black line), and the SW cross interaction *u*_12_(*r*), plotted for all ε_12_ investigated in this work (in the legend). The HS interaction *u*_22_(*r*) is not shown.

Our mixture consists of hard-sphere two-Yukawa particles and hard spheres, henceforth labeled 1 and 2 respectively, with the same core diameter σ. Beyond the hard core, the HSTY interaction is given by
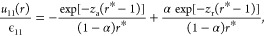
1where r* = *r*/σ; ε_11_ is the potential minimum at σ; *z*_a_, and *z*_r_ are the inverse range of attraction and repulsion, respectively; α is the ratio of strength of repulsion to attraction. The range of the attractive well extends up to
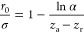
2i.e., the zero of *u*_11_(*r*). In particular, we choose *z*_a_ = 10, *z*_r_ = 0.5, and α = 0.1, and therefore, *r*_0_ ≃ 1.242σ. With this parametrization, *u*_11_(*r*) corresponds to the HSDY1 case studied in ref (18). The cross interaction *u*_12_(*r*) is provided by a SW attraction of depth ε_12_ (with the ratio ε_12_/ε_11_ varying between 0.01 and 1) and range *r*_0_. With this prescription, the HSTY and SW interactions have the same attractive range. In Figure 1, we plot the shapes of *u*_11_(*r*) and *u*_12_(*r*) for all the values of ε_12_ investigated in this work. We take σ and ε_11_ as units of length and energy, respectively. Then, the density is given in units of σ^–3^ while the temperature is measured in units of ε_11_/*k*_B_, where *k*_B_ is the Boltzmann constant.

We carry out canonical-ensemble Monte Carlo (MC) simulations of a mixture composed of *N*_1_ HSTY particles and *N*_2_ HS particles, with *N* = *N*_1_ + *N*_2_ = 2048, enclosed in a cubic box with periodic boundary conditions to reduce surface effects. The concentration *N*_1_/*N* of HSTY particles is denoted χ. Before moving to the mixture, we have studied a one-component system of 500 HSTY particles, to fix reference thermodynamic conditions for the clustering of the pure fluid. Thereby, we have set ρ = 0.05, *T* in the range 0.20–0.25, whereas χ varies over the full range of relative concentrations. In our simulations, *u*_11_(*r*) is truncated at half the box length (10.77σ for the pure HSTY fluid and 17.23σ for the mixture). The chosen density is high enough to allow for cluster formation at low temperatures, but still sufficiently low to prevent packing effects from playing any role. In the selected temperature range, the pure HSTY fluid undergoes the transformation from a weakly inhomogeneous state to a deeply clustered state.

The particles are initially distributed at random (*T* = *∞*); then, the system is quenched at a given temperature *T* and equilibrated. From our experience on self-assembling mixtures we know that a huge number (higher than 10^8^) of MC cycles is typically required to properly equilibrate the system at low temperatures. Therefore, we have optimized our MC code in the attempt to make the numerical algorithm converge faster. In particular, we have implemented two distinct values for the maximum random displacement (mrd) of a particle, so as to account for the possible existence of two different (intracluster and intercluster) length scales in the system. The two mrd values are adjusted during the equilibration run until the acceptance of translational MC moves is around 30% for the longer moves and 80% for the shorter ones. This gives mrd values of ≈0.1σ and ≈0.01σ, respectively. Furthermore, we have introduced swapping moves, whereby the positions of two randomly chosen HSTY and HS particles are exchanged. For both moves, acceptance is decided based on the standard Metropolis test. The schedule of all moves, the canonical moves and the new ones, is such that detailed balance holds exactly at each step.

The structure of the mixture is investigated by computing the radial distribution function *g*_*ij*_(*r*) and the structure factor *S*_*ij*_(*k*), with *i* and *j* labeling the two different species. Structure factors are calculated as Fourier transforms of *g*_*ij*_(*r*), according to the formula^37^
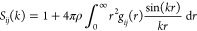
3

To gain knowledge on the self- and cross-correlations of global variables, we also compute the Bathia–Thornton number–number, concentration–concentration, and number-concentration structure factors of the mixture, defined as^55,56^
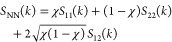
4
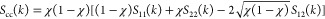
5
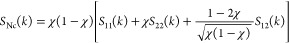
6respectively.

Aggregates are characterized through a cluster analysis performed by the Hoshen–Kopelman algorithm.^57^ According to our convention, two particles (of whatever species) are bonded if they are separated by less than *r*_0_, i.e., if the particles fall within the range of the SA/SW potential. Following refs^18 and 58^, the CSD is taken to be
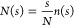
7where *n*(*s*) is the average number of clusters with size *s* in a single configuration. The distribution *N*(*s*) is normalized in such a way that ∑_*s*_*N*(*s*) = 1. In our study, the distribution of cluster sizes is computed by averaging over 1000 equispaced configurations extracted from the last part of the production run.

## III Pure HSTY Fluid

**Figure 2:**
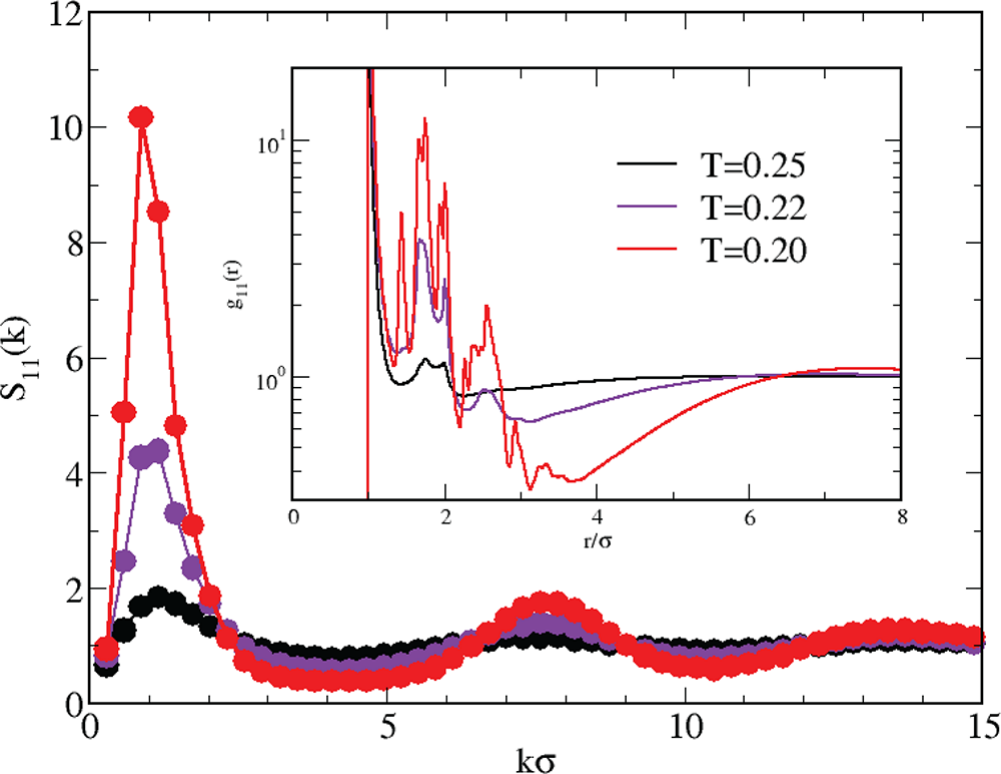
Structure factor and radial distribution function (inset) of the pure HSTY fluid for ρ = 0.05 and three different temperatures (in the legend).

Information about the structure attained by the HSTY fluid long after the initial quench can be gained from the profile of the total structure factor *S*_NN_(*k*) ≡ *S*_11_(*k*), reported in Figure 2. For *T* = 0.25, the height of its low-*k* peak, located at *k*_0_σ ≈ 1, attains a value ≈1.8, which would suggest, according to the heuristic criterion of refs (18 and 19), that the fluid exhibits intermediate-range order. This condition preludes to the subsequent development of more structured aggregates for *T* = 0.22, where indeed *S*_11_(*k*_0_) rises to ≈4.2, i.e., above the clustering threshold of ≈3. Based on the structural evidence, for *T* = 0.20 [where *S*_11_(*k*_0_) exceeds 10] clusters appear to be well structured. As for the real-space structure, we report in the inset of Figure 2 the radial distribution function *g*_11_(*r*) for the same three temperatures. While for *T* = 0.25 the system is little structured, the multiple solid-like peaks of *g*_11_(*r*) for *T* = 0.22, and even more so for *T* = 0.20, indicate a high degree of order in the arrangement of particles around a reference particle, i.e., within the linear extent of a cluster. The subsequent long-range oscillations in the spatial correlations for *r* ≳ 4σ are another well-known indication that a modulated phase is taking over in the fluid.

**Figure 3:**
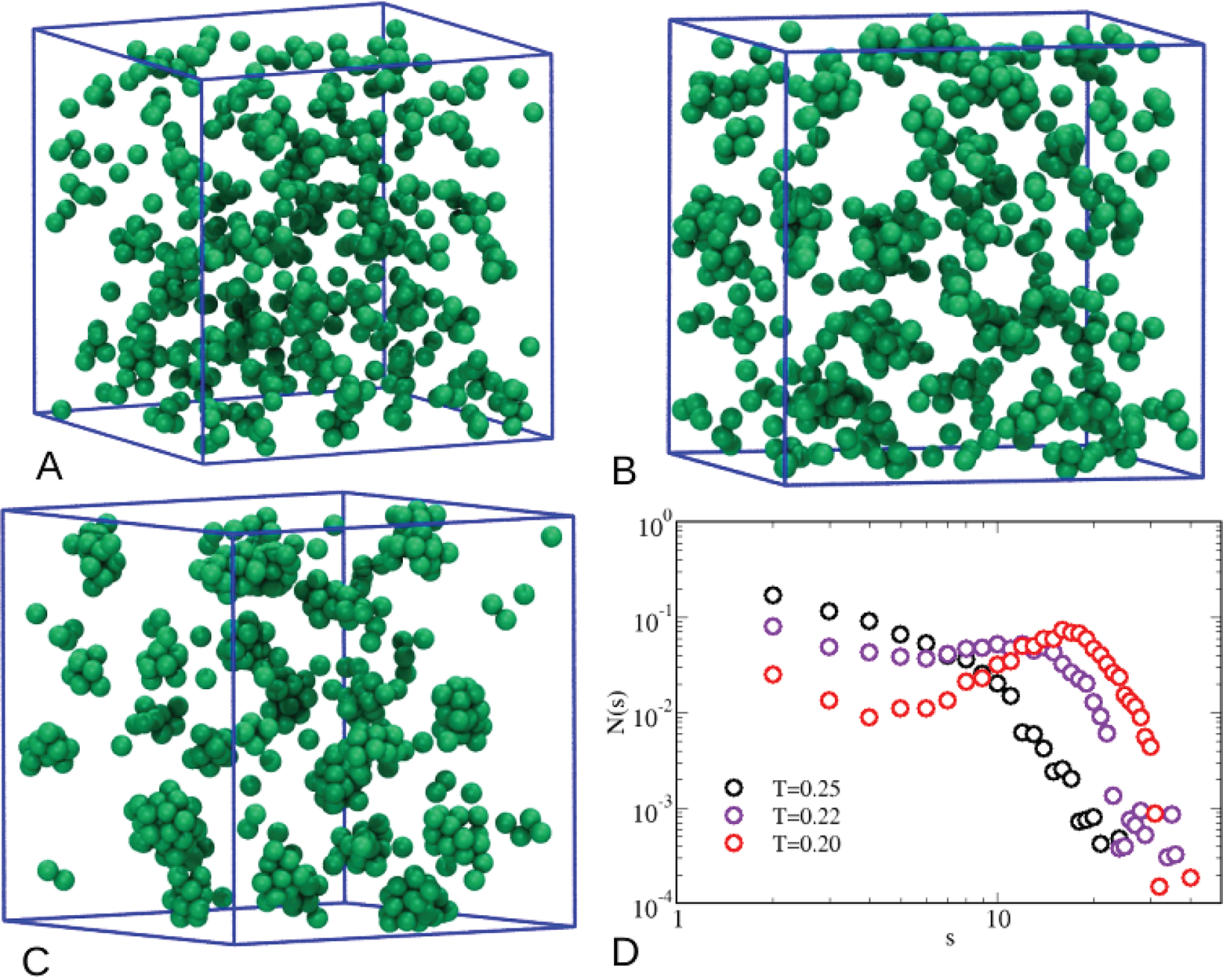
Microscopic configurations of the pure HSTY fluid. (A) *T* = 0.25; (B) *T* = 0.22; (C) *T* = 0.20; (D) CSD at the same temperatures.

The local arrangement of the HSTY fluid is better elucidated by a visual inspection of microscopic configurations, see Figure 3. For *T* = 0.25 (A), in the wake of development of intermediate-range order, the fluid appears to be overall homogeneous, even though local inhomogeneities are occasionally seen. Instead, for *T* = 0.22 (B), the tendency of particles to aggregate is more evident, with clusters starting to form in the system. For *T* = 0.20 (C), very few particles are isolated and the cluster shape is approximately spherical. Information on the cluster structure can be gained from the CSD, plotted in Figure 3D: this decays monotonically for *T* = 0.25, certifying that the system almost behaves as a dispersed fluid. For *T* = 0.22 a shallow peak develops around *s* = 15, witnessing the presence of clusters with this typical size. The sign of clustering becomes definitely sharp at *T* = 0.20, where a distinct peak is visible in the CSD at *s* ≈ 20.

To summarize this section, we have a coherent structural and microscopic picture showing that in the HSTY fluid with density ρ = 0.05 clusters are absent for *T* = 0.25; they start appearing for *T* = 0.22 and are fully developed for *T* = 0.20. In another study of the same model,^18^ at about twice the density analyzed here, the fluid for *T* = 0.25 was found in a clustered state, suggesting that the characteristic temperatures marking the onset of the various aggregation stages—intermediate-range order first and clustered state afterward, possibly evolving into an arrested state along the so-called λ-line—show a shallow parabolic trend as a function of density, which is reminiscent of the liquid–vapor binodal in fluids with short-range attraction.

## IV Equimolar HSTY–HS Mixture

Having clarified the behavior of the HSTY fluid, we move to the binary mixture obtained from the former by replacing half of the particles with hard spheres (i.e., χ = 0.5). We choose thermodynamic conditions—ρ = 0.05 and *T* = 0.25—such that, as shown in the previous section, no stable clusters are present in the HSTY fluid. Our purpose is to establish whether the presence of another species, able to provide a bridge between two nearby HSTY particles, is sufficient to promote cluster formation. In principle, this is one out of several scenarios that could hypothetically arise once the balance between attraction and repulsion in the mixture moves in favor of the former; other two plausible outcomes, related to the strength of cross attraction ε_12_, are that either the mixture stays almost homogeneous as the pure HSTY fluid or, at the opposite end, that the mutual attraction is now strong enough to overcome the competition with repulsion, thus giving rise to liquid–vapor separation. In this regard, it seems unlikely that demixing can occur, because ruled out by the 1–2 attraction.

**Figure 4:**
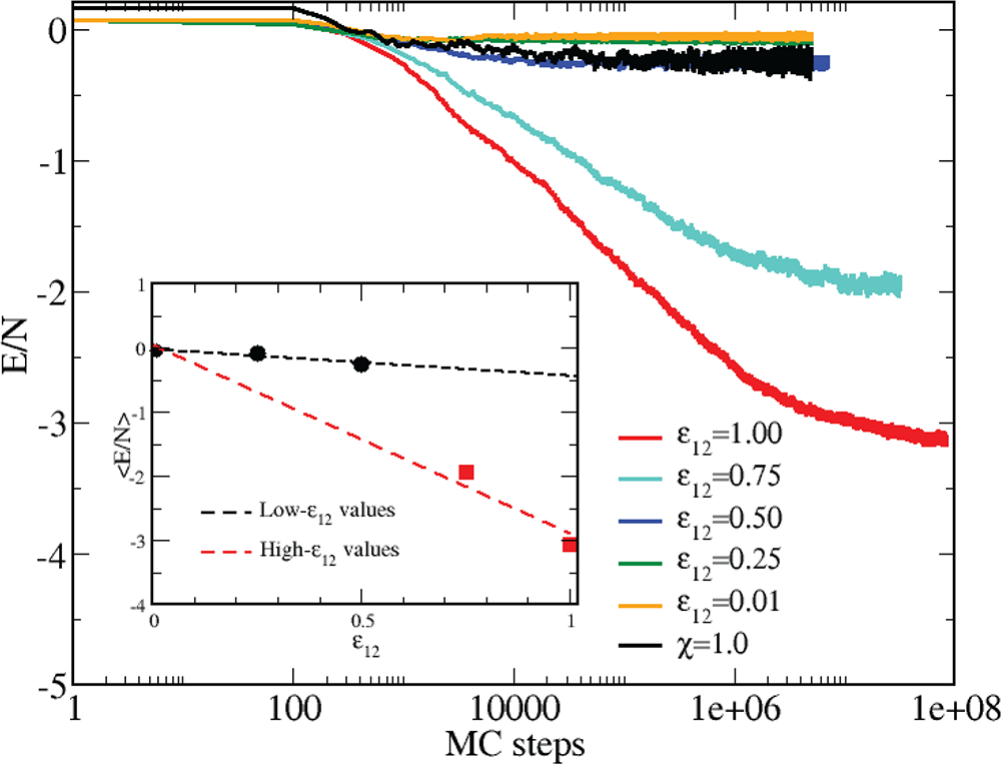
Equimolar HSTY–HS mixture for *T* = 0.25. Potential energy per particle vs MC cycles for different ε_12_, in the legend; results for the pure HSTY fluid are also shown, for comparison. Inset: asymptotic values (symbols) vs ε_12_ are fitted by two different straight lines.

**Figure 5:**
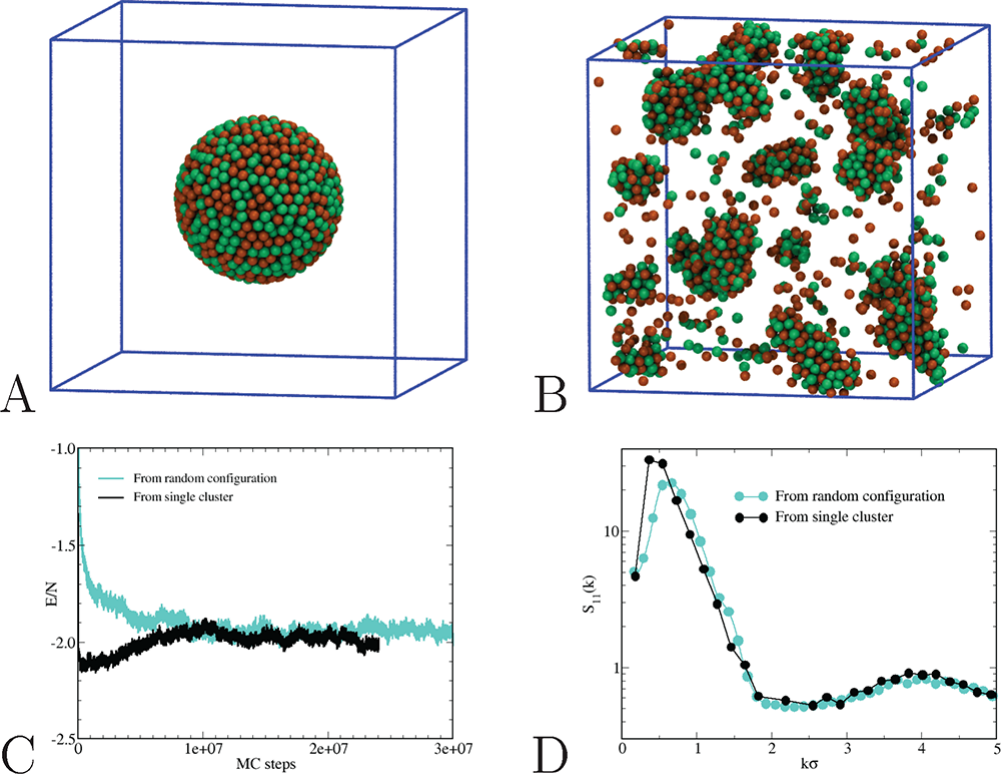
Equimolar HSTY–HS mixture for *T* = 0.25 and ε_12_ = 0.75. (A) Big-cluster initial configuration, containing 2048 randomly dispersed HSTY and HS spheres. (B) After 2.5 × 10^7^ MC moves, the initial cluster is reduced in fragments, now resembling the cluster fluid obtained from a fully homogeneous initial configuration; see Figure 9B below. After equilibration, the potential energy fluctuates around a fixed value, which is roughly independent of the initial configuration (C). Interestingly enough, the initial energy of the system in the big cluster is lower than in the final cluster-fluid configuration; hence, it is the relevant entropy gain achieved by forming many smaller clusters that ensures that the cluster fluid is more stable than the big cluster. Also the final structure factors are similar in the two runs (D), but for a small mismatch in the position and height of the low-*k* peak.

To ascertain this issue we have simulated equimolar mixtures for values of ε_12_ in the range 0.01–1 (in units of ε_11_). Results for the potential energy are collected in Figure 4: as a preliminary observation, the equilibration time strongly depends on ε_12_, ranging from 10^6^ MC cycles for ε_12_ = 0.01 to 10^8^ or more MC cycles for ε_12_ = 1. To check whether equilibrium has been reached—especially for the deepest attractions, ε_12_ > 0.50—we have performed additional simulations for ε_12_ = 0.75 and ε_12_ = 1, using a big spherical cluster encompassing all particles as initial configuration, see Figure 5A. As for ε_12_ = 0.75, we have verified that the final state of the system along this route (in terms of potential energy, structural, and microscopic appearance, see Figure 5B–D) matches with the previous one, indicating that equilibrium has eventually been attained. On the other hand, for ε_12_ = 1, the time needed for the decay of the big-cluster configuration is much longer than we could achieve in our analysis. Our conclusion, also supported by the slow energy drift in Figure 4, is that for ε_12_ = 1 the system is still slowly approaching equilibrium, even though this fact would have no serious impact on the considerations presented below.

Turning back to the behavior of the potential energy per particle in Figure 4, it appears that for ε_12_ ≤ 0.50 the mixture behaves similarly to the HSTY fluid of same *T*; i.e., the system quickly attains equilibrium and the final energy is between −0.05 and −0.25. However, when ε_12_ is increased from 0.50 to 0.75, the scenario changes drastically, with the energy now exhibiting a sharp drop until reaching a final value of ≈−2 after about 10^7^ MC steps. Upon further increasing the attraction strength to ε_12_ = 1, the energy approaches ≈−3 after about 5 × 10^7^ MC steps. In particular, we see that the rate of (absolute) energy increase grows abruptly as ε_12_ overcomes 0.50. This can be appreciated in the inset of Figure 4, where the asymptotic energies are reported as a function of ε_12_: the energy values can be grouped in two sets (i.e., up to ε_12_ = 0.50 and from ε_12_ = 0.75 onward) which are fitted by different straight lines, in this way corroborating the idea that the crossover between the two regimes follows a threshold-like behavior.

**Figure 6:**
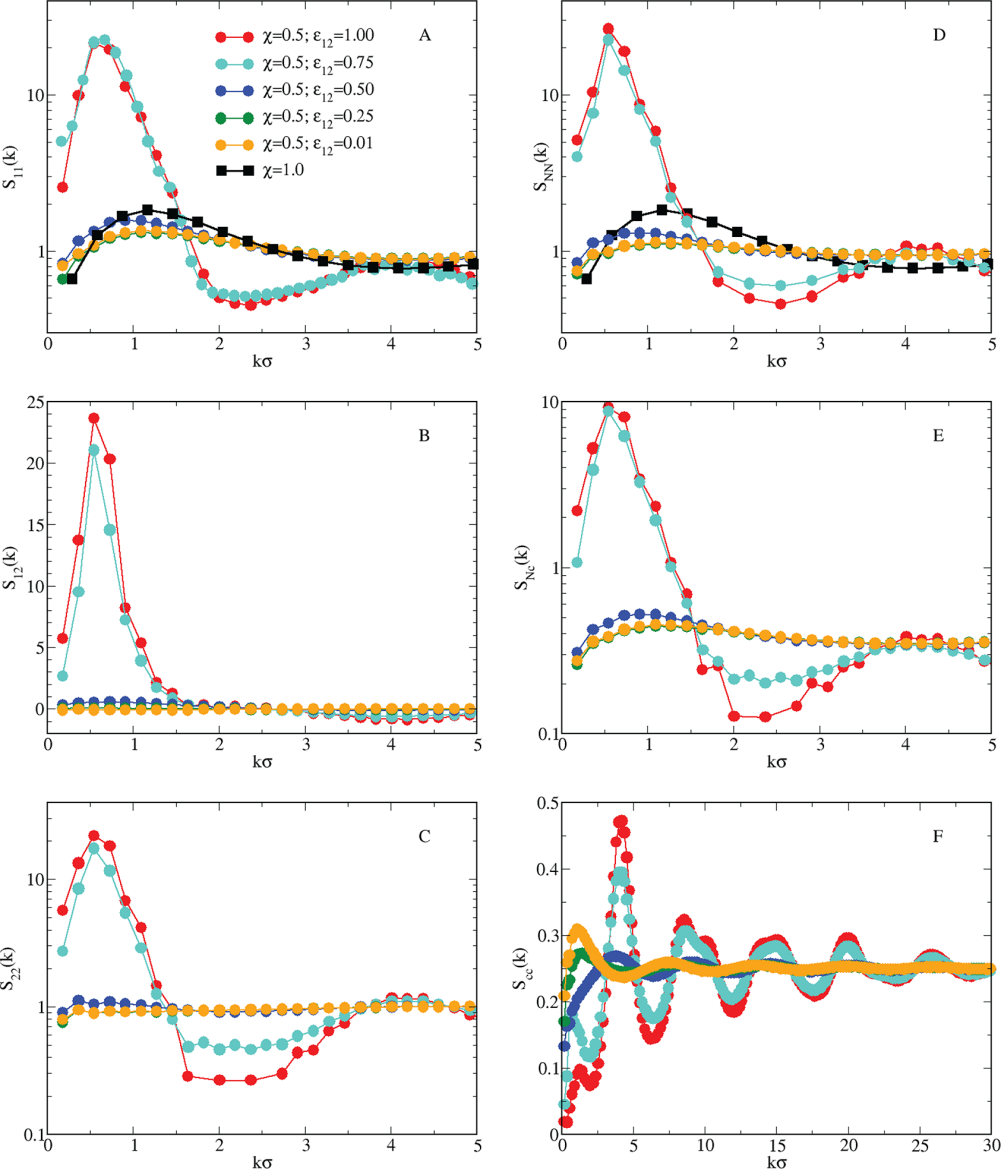
Equimolar HSTY–HS mixture for *T* = 0.25. (A) *S*_11_(*k*); (B) *S*_12_(*k*); (C) *S*_22_(*k*); (D) *S*_NN_(*k*); (E) *S*_Nc_(*k*); (F) *S*_cc_(*k*). Values of ε_12_ are shown in panel A, with the same colors applying for all panels. The pure HSTY case is also reported in panels A and D for comparison. Note the semilogarithmic scales in panels A, C, D, and E.

The evolution of potential energy with ε_12_ suggests that the system exhibits some form of aggregation for ε_12_ > 0.50, even though we cannot yet say whether it is liquid–vapor separation or clusterization that occurs. To clarify this point, we analyze in Figure 6A the partial structure factor *S*_11_(*k*) of HSTY particles: we see that, upon increasing ε_12_, the *k* → 0 limit of *S*_11_(*k*) does not change significantly; on the other hand, for ε_12_ > 0.50, *S*_11_(*k*_0_) jumps over 10, indicating that clusterization, rather than liquid–vapor separation, takes place in the system for sufficiently strong cross attraction. Correlations involving species 2 are shown in panels B and C of Figure 6, but they hardly add anything new to what we have already learned from *S*_11_(*k*), since *S*_12_(*k*) and *S*_22_(*k*) also show a remarkable increase of the low-*k* peak for ε_12_ > 0.50. Global variables, expressed according to eqs 4–6, are reported in panels D–F for the same values of ε_12_: the total structure factor *S*_NN_(*k*) (D) is similar to *S*_11_(*k*), both in shape and height of the peaks, confirming that the indications provided by the structure factor of the HSTY species alone hold for the mixture too. A similar trend is also observed in *S*_*Nc*_ (E), whereas *S*_*cc*_ (F), behaves differently: in this case, besides the development of the low-*k* peak, we observe an increasing structuring with ε_12_, as indicated by the multiple peaks found for ε_12_ > 0.50. Therefore, significant concentration fluctuations are found over a wide range of *k* values, as can be expected in a cluster-forming system, due to the spatial rearrangement of particles inside the aggregates.

**Figure 7:**
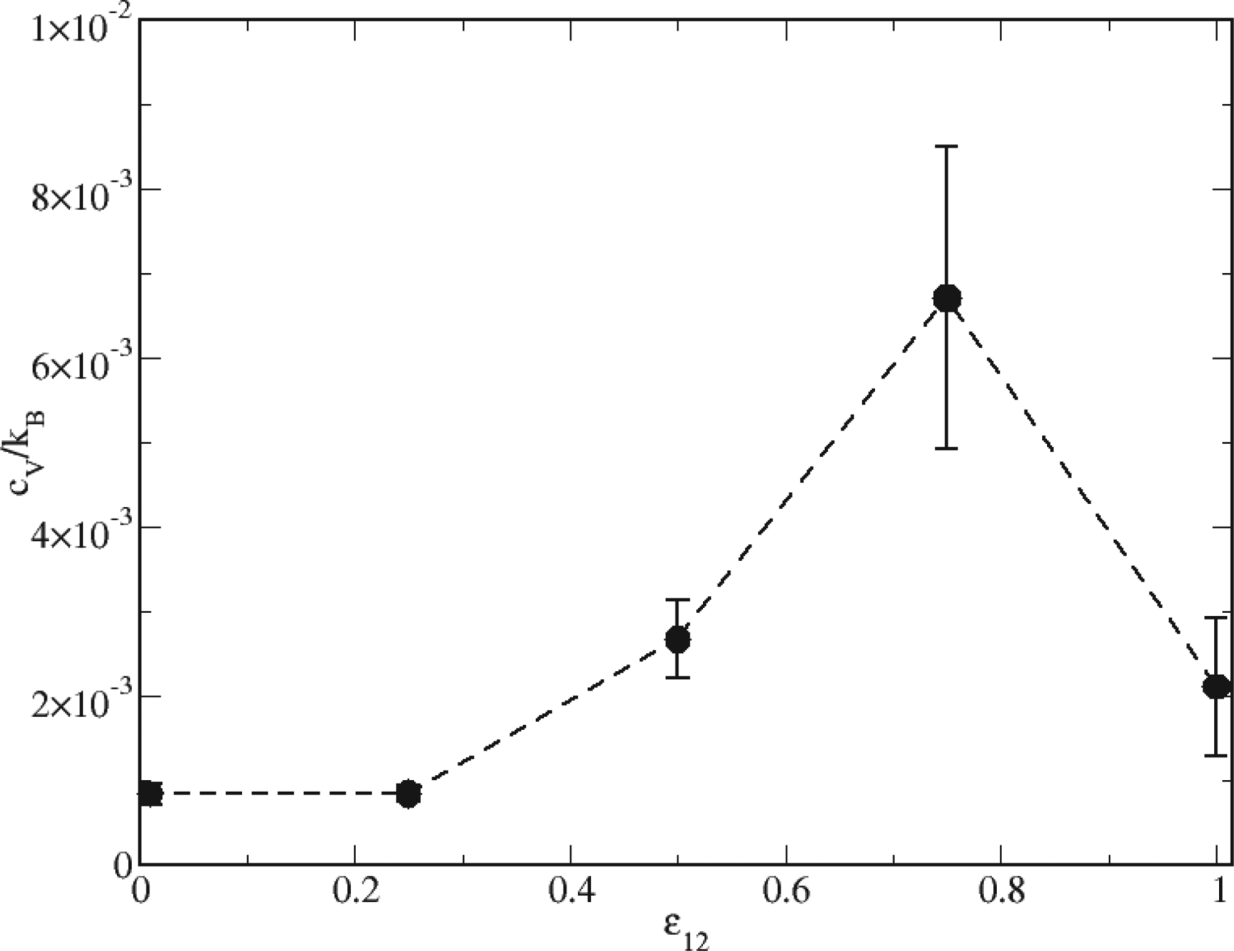
Equimolar HSTY–HS mixture for *T* = 0.25. *c*_V_/*k*_*B*_ vs ε_12_. The error bars represent maximum dispersions from the mean as computed from 10 independent runs.

To complete our thermodynamic/structural survey, we mentioned in the Introduction that a maximum in the specific heat at constant volume, *c*_V_, provides another reasonable indication of the onset of clustering.^43^ In particular, the specific heat is related to energy fluctuations according to the expression^37^
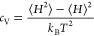
8(where *H* is the Hamiltonian of the system) and indeed such fluctuations become large when clusters form and disrupt with similar probability. In Figure 7, *c*_V_ is reported as a function of ε_12_ at constant *T* = 0.25. It clearly emerges that the observed behavior corroborates our previous finding: *c*_V_ shows a maximum for ε_12_ = 0.75, where, according to internal energy (see Figure 4) and structure factors (see Figure 6), clusters show up for the first time. For ε_12_ = 1 clusters are already formed and quite stable, therefore energy fluctuations are less marked and in turn *c*_V_ attains smaller values.

**Figure 8:**
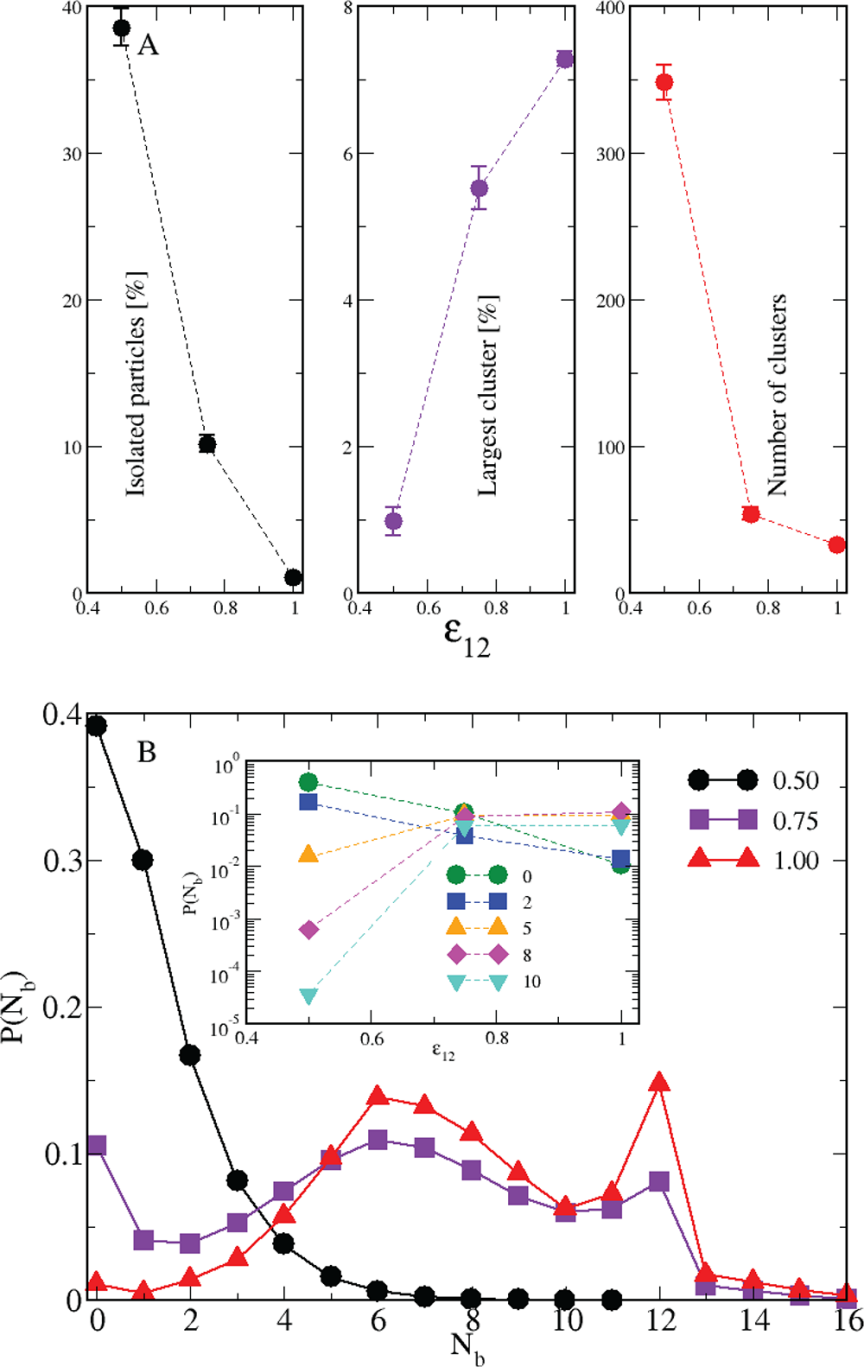
Equimolar mixture for *T* = 0.25. (A) Number of isolated spheres (left), largest cluster size (middle), and total number of clusters (right) for various ε_12_ values. Standard deviations are also shown. (B) Probability distribution of the number of bonds for a number of ε_12_ values, in the legend. In the inset, the same data are plotted vs ε_12_ for various *N*_b_, in the legend.

Once having ascertained that a second species is able to promote the cluster formation under proper conditions, we have computed a number of properties to characterize the structure of aggregates, see Figure 8A: the number of isolated particles monotonically decreases with increasing ε_12_ (left), signaling that aggregates encompass more and more particles; concurrently, the largest cluster size increases (middle) and the total number of clusters decreases (right), indicating that aggregates appear and grow in size with increasing ε_12_. This picture is reflected in the probability distribution of the number of bonds, plotted in Figure 8B. Here the particles are classified according to the number of bonded “neighbors”: the monotonic decay for ε_12_ = 0.5 is replaced by a trimodal distribution for ε_12_ = 0.75, with peaks at *N*_b_ = 0, 6, and 12, respectively associated with isolated particles, outer cluster particles, and inner cluster particles. In particular, the peak at *N*_b_ = 12 (more enhanced for ε_12_ = 1) strongly points to a liquid-like (if not even fcc-like) ordering inside clusters. The comparatively higher number of bonds formed for larger values of ε_12_ can be better appreciated in the inset of Figure 8B: here the probability to form more than two bonds is remarkably low for ε_12_ = 0.5, whereas the trend is reversed for ε_12_ = 0.75 and, even more so, for ε_12_ = 1, where 8 and 10 bonds per particle occur with a rather high frequency.

**Figure 9:**
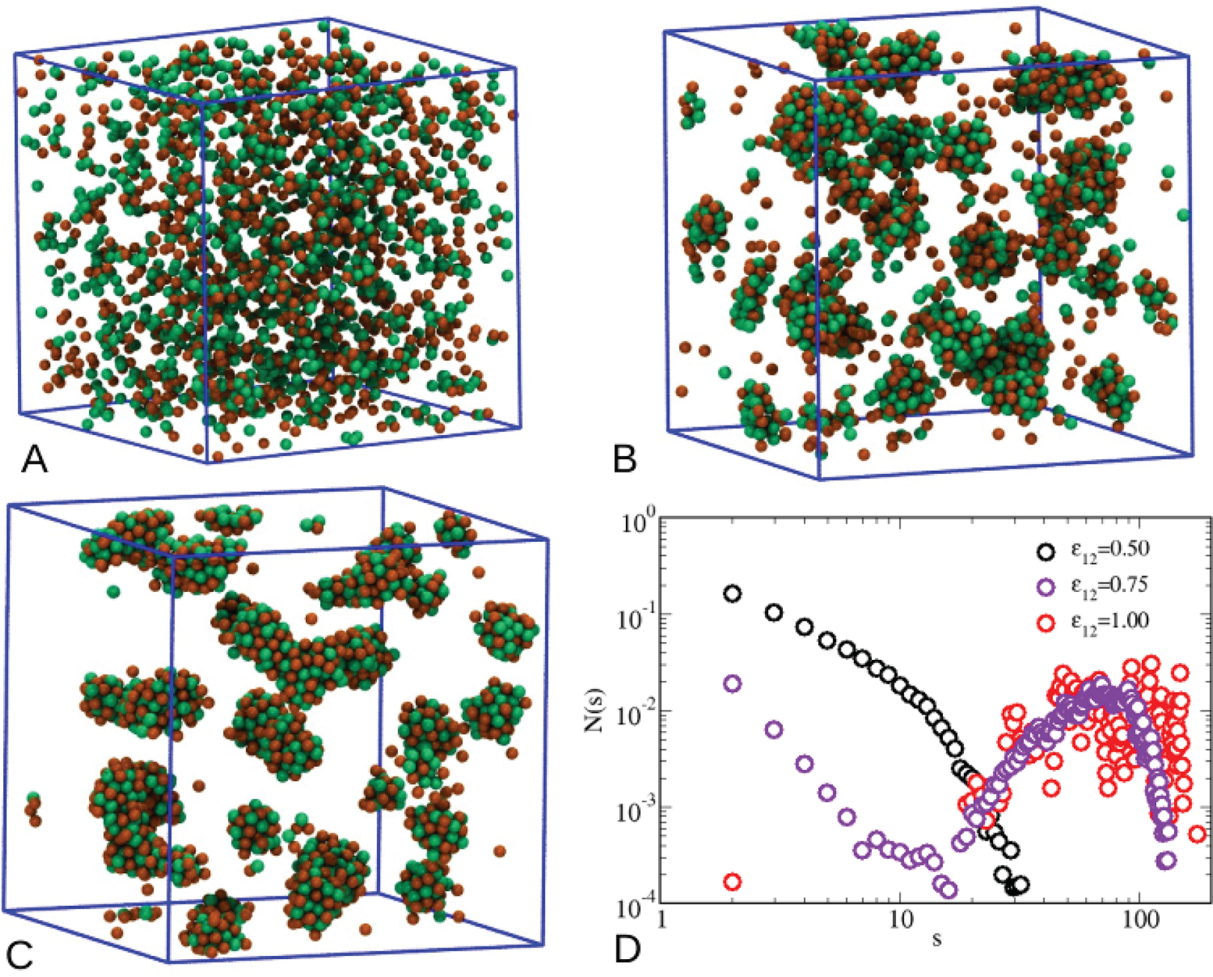
Microscopic configurations for the equimolar mixture at *T* = 0.25 (HSTY, green; HS, orange). (A) ε_12_ = 0.5; (B) ε_12_ = 0.75; (C) ε_12_ = 1; (D) CSD for the same values of ε_12_.

The above picture is completed by the microscopic configurations reported in Figure 9: as ε_12_ grows from 0.50 (A) to 0.75 (B), up to 1 (C), clusters become progressively more structured. In analogy with the behavior of the pure HSTY fluid upon cooling (Figure 3), for ε_12_ = 0.75 and 1, the shape of the clusters is approximately spherical, although in the latter case nonspherical aggregates also exist. Under these conditions, very few isolated particles appear in the box, due to a strong HSTY–HS interaction. A closer look at Figure 9C shows that cluster particles are instead tightly packed for ε_12_ = 1, similar to what occurs in the solid-like nuclei developing in a supercooled vapor or liquid which is about to crystallize.^59^ Indeed, a high degree of order in the arrangement of particles within a cluster is implied by the behavior of *g*_11_(*r*) (not shown here), which displays multiple solid-like peaks akin to those observed in the inset of Figure 2 for *T* ≤ 0.22. More refined investigations of the liquid–solid transition inside clusters, like those described in ref (60), are deferred to future studies. The cluster-size statistics (computed by making no difference between the species) is reported in Figure 9D: for ε_12_ = 0.50 the CSD is a monotonically decreasing function of the cluster size *s*, a profile compatible with a dispersed fluid. On the contrary, for ε_12_ = 0.75 the CSD has a well-definite maximum around *s* = 60, indicating this preferred cluster size. In addition, we observe a non-negligible fraction of larger clusters comprising up to 100 particles. For ε_12_ = 1, we see multiple peaks in the CSD, suggesting the existence of as many preferential cluster sizes, although the distribution is centered around the same size as for ε_12_ = 0.75. In addition, we see no sign of a percolated structure, in spite of the existence of large aggregates with up to 200 particles. Overall, the distributions reported in Figure 9 closely resemble those in Figure 3, suggesting that the strength of cross attraction plays a role similar to inverse temperature in a HSTY fluid.

Summing up, a sufficiently large ε_12_ can sustain clusters in an equimolar HSTY–HS system, notwithstanding their absence in the pure HSTY fluid of same density and temperature. The same conclusion was already drawn in ref (54) but using mutually attractive spheres as foreign species. Our results demonstrate that even particles that do not attract each other are able to provide the extra attraction needed by HSTY particles to undergo clusterization.

To rationalize our evidence, we turn to the interpretation of clustering given in refs (18 and 19). Therein it is argued that the clustering threshold in HSTY systems falls upon (roughly) the same thermodynamic conditions for which a modified HSTY fluid with the long-range repulsion switched off would phase-separate into liquid and vapor. We hereafter refer to this modified HSTY fluid [i.e., with *u*_11_(*r*) truncated at *r*_0_] as the “reference attractive” HSTY fluid. In other words, adding an appropriate long-range repulsion on top of a purely attractive tail breaks the liquid–vapor mixture into separate, noninteracting fragments (the clusters). A similar scenario has been described in regard to spinodal decomposition in ref (23), where the behavior of a fluid interacting via a generalized Lennard-Jones plus a repulsive-Yukawa tail was investigated in the regime where arrested states hold the stage. If the same argument applies for the present mixture, then for *T* = 0.25, χ = 0.5, and ε_12_ = 0.75 the liquid–vapor binodal line of the reference attractive HSTY–HS mixture will lie above that of the reference attractive HSTY fluid. Accordingly, a measure of the overall amount of attraction (i.e., the “glue” keeping particles together) will be larger for the mixture than for the pure HSTY fluid alone.

**Figure 10:**
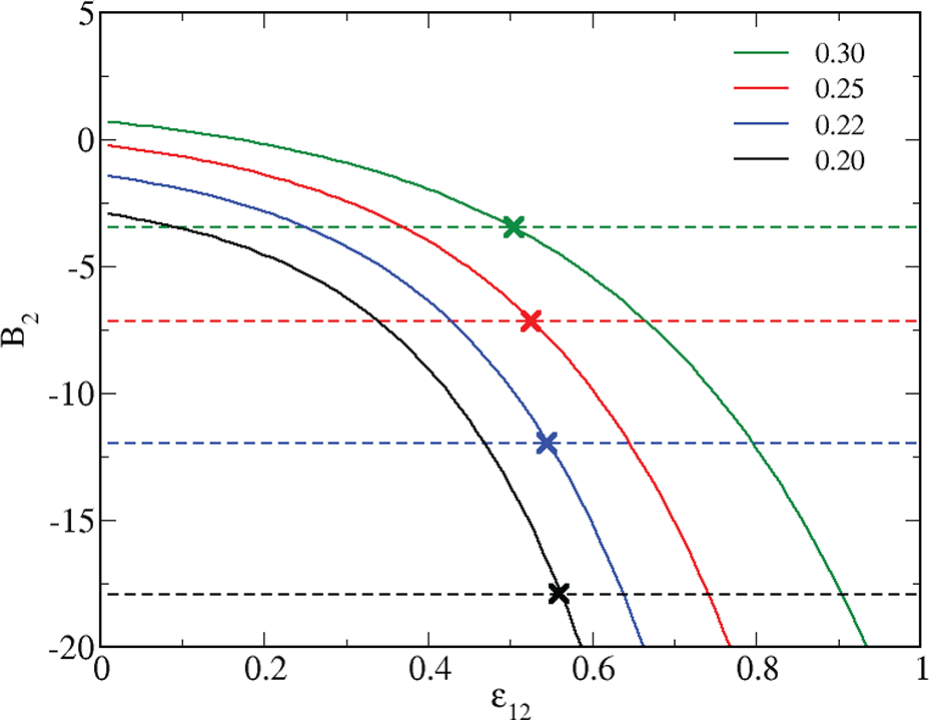
Second virial coefficient of the reference attractive fluids for the equimolar mixture (full lines) and the pure HSTY (dashed lines) vs ε_12_ for various temperatures, in the legend. Intersections are marked by crosses.

To check this conjecture, we have employed the second virial coefficient *B*_2_, which provides an integrated measure of the strength of interactions in the system. The virial coefficient of the reference attractive mixture is^61^
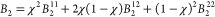
9with
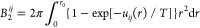
10for *ij* = 11 or 12, and *B*_2_^22^ = 2*πσ*^3^/3. Only the first term on the r.h.s. of eq 9 (with χ = 1) needs to be considered for the reference attractive HSTY fluid. In Figure 10 we plot *B*_2_ and *B*_2_^11^, for χ = 0.5 and different temperatures, as a function of ε_12_: for a given *T*, the intersection between *B*_2_ and *B*_2_^11^ falls at
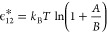
11with
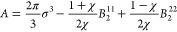
12and
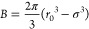
13

For the temperatures in Figure 10, we have the pairs [*T*, ε_12_^*^] = [0.20, 0.5621], [0.22, 0.5465], [0.25, 0.5272], and [0.30, 0.5032], each marked by a cross in the figure. In particular, for *T* = 0.25, the reference attractive mixture will be more attractive than the reference attractive HSTY fluid for ε_12_ > ε_12_^*^ = 0.5272 (see red lines and cross). Accordingly, the liquid–vapor binodal of the former falls above the binodal line of the latter for ε_12_ > ε_12_^*^, while lying below otherwise. This prediction is in gratifying agreement with results shown in Figures 4–9, confirming the validity of our approach: for *T* = 0.25 the equimolar mixture with ε_12_ = 0.50 is unable to sustain cluster formation, similar to the HSTY fluid; conversely, for ε_12_ = 0.75, the clustering tendency of the mixture will be enhanced with respect to the HSTY fluid.

**Figure 11:**
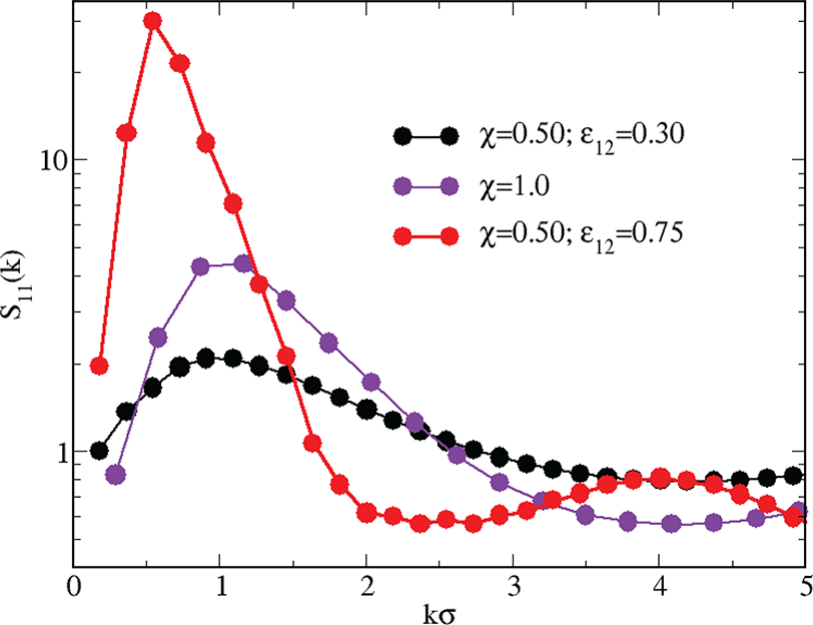
Equimolar mixture for *T* = 0.22. *S*_11_(*k*) for ε_12_ = 0.30 and 0.75. Results for the pure HSTY fluid of the same density and temperature are also reported for comparison.

We may wonder whether the same comparison also applies in reverse, so that clusterization is preempted in the mixture for the same density and temperature where clusters occur in the pure HSTY fluid. To verify this hypothesis, we take *T* = 0.22, where clusters are present in the pure HSTY fluid (see Figure 2). At this temperature, the crossing of *B*_2_ lines occurs for ε_12_^*^ = 0.5465 (see Figure 10, blue lines and cross). Structural data are presented in Figure 11 for ε_12_ = 0.30 and 0.75, i.e., two values on opposite sides of 0.5465. While the height of the low-*k* peak is ≈4 in the pure HSTY fluid, the same peak falls below 2 in the mixture with ε_12_ = 0.30, meaning that with a proper choice of ε_12_ (to be discriminated on the basis of the *B*_2_ criterion) clustering can be suppressed. On the other side of the threshold, i.e., for ε_12_ = 0.75, the attraction is so intense that clusterization is strongly enhanced [*S*(*k*_0_) ≈ 30].

To sum up, the crossing of the *B*_2_ vs ε_12_ lines falls in the narrow interval ε_12_ ≈ 0.50–0.56 in the *T* range 0.20–0.30, where the pure HSTY fluid experiences the crossover from a rather homogeneous fluid to a strongly structured cluster fluid. Our method provides a viable means to tune the onset of clustering in the equimolar HSTY–HS mixture; this tuning acts in both directions, namely enhancing or depressing the tendency to clusterization by just increasing or decreasing the strength of cross attraction at a given temperature. We have thus provided another assessment of the clustering picture proposed in refs (18, 19, and 23), according to which clusters develop for the same thermodynamic conditions where the reference attractive fluid undergoes liquid–vapor separation.

Our analysis also confirms that *S*(*k*_0_) ≈ 3 is a sensitive indicator of the onset of clustering. As we move around this value (in the present case, in the temperature range 0.22–0.25, see Figure 2), clusters can be reversibly created or destroyed by adjusting ε_12_ on the basis of the relative balance in *B*_2_ between the (attractive) mixture and HSTY fluid. However, this tuning no longer works when we penetrate too much in the clustering region. Indeed, for *T* = 0.20 [where *S*(*k*_0_) ≈ 10 in the pure HSTY fluid, see Figure 2] we find *S*(*k*_0_) ≳ 12 for ε_12_ = 0.75 and *S*(*k*_0_) ≳ 6 for ε_12_ = 0.30. Therefore, a change in ε_12_ will certainly affect the quality of the clustered state, but with no possibility to recover homogeneity.

## V Changing the Relative Concentration

**Figure 12:**
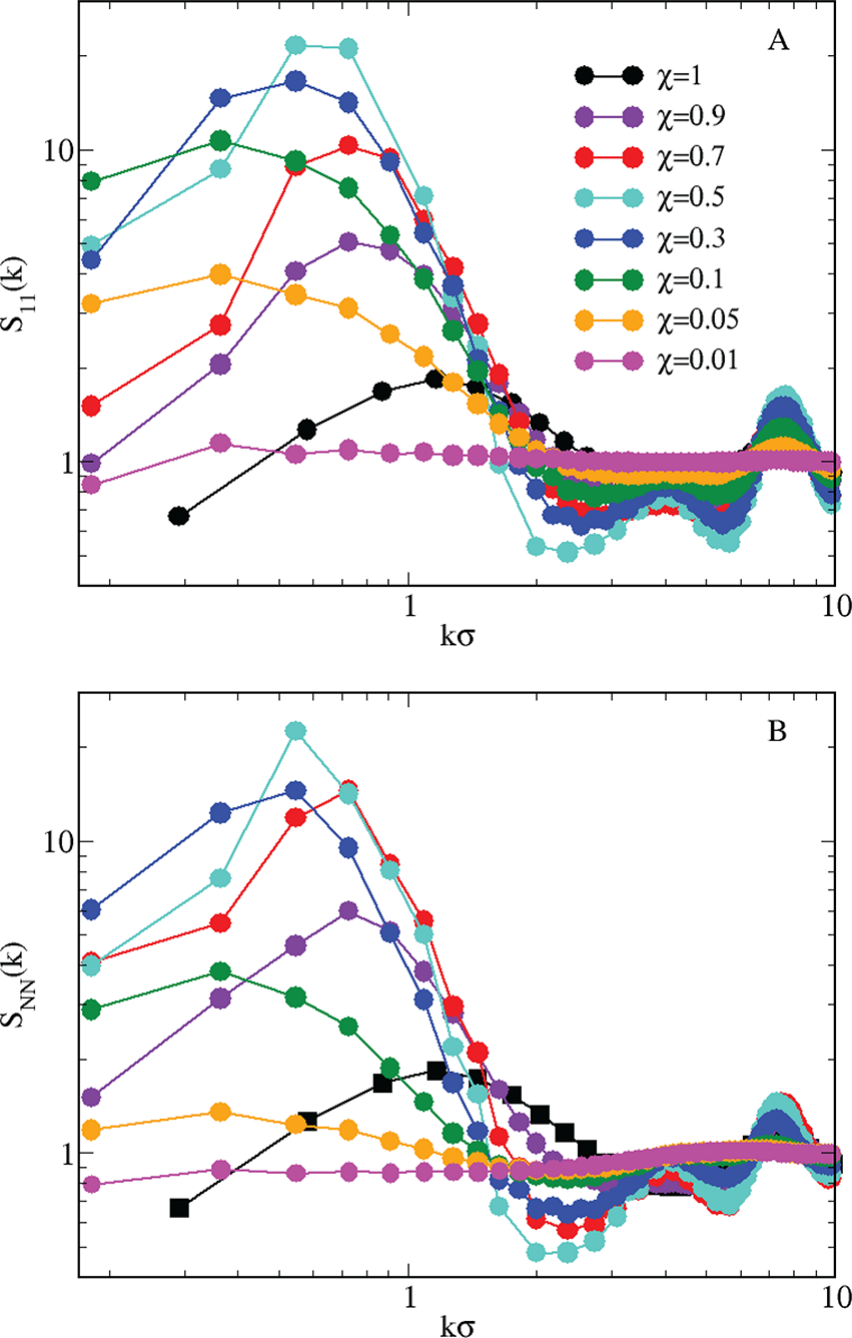
Mixture for *T* = 0.25 and ε_12_ = 0.75. (A) *S*_11_(*k*); (B) *S*_NN_(*k*). The whole range of HSTY concentrations is shown; see the legend.

Finally, we elucidate the effect of varying the relative concentration of the species on the formation of clusters. We set *T* = 0.25 and ε_12_ = 0.75 (allowing for the presence of clusters in the equimolar mixture, as shown in the previous section) and span the whole range of HSTY concentrations. The behavior of *S*_11_(*k*) is shown in Figure 12A, where, for the sake of comparison, we have also included *S*(*k*) of the pure HSTY fluid. We see that *S*_11_(*k*_0_) > 3 in almost all cases; in particular, *S*_11_(*k*_0_) falls between 4 and 5 for both χ = 0.05 and χ = 0.9, while rising above 10 between these extremes. As a result, at the given conditions of temperature and density, changing the concentration will only moderately affect the clustered nature of the mixture, with the only exception being χ = 0.01, where clusters are apparently absent. In particular, we can induce clustering in a fluid of hard spheres by replacing a fraction as low as 5% of HS particles with HSTY particles. Moreover, compared to the pure fluid, the main peak of *S*_11_(*k*) is more pronounced and better resolved; at the same time, *k*_0_ moves closer and closer to zero as hard spheres become progressively dominant in the mixture, pointing to the presence of increasingly large clusters. Therefore, the inclusion of HS particles invariably gives rise to stabilization of the clustered state, with the strongest effect near equimolarity, as demonstrated by the largest value of *S*(*k*_0_) attained for χ = 0.3 and 0.5. The tendency of HSTY particles to form clusters acts as a dragging mechanism for the structural properties of the whole mixture, as can be appreciated from the similarity between *S*_11_(*k*) and the total structure factor *S*_NN_(*k*) in Figure 12B. Some discrepancy only emerges for low χ, where indeed a large number of HS particles stay isolated, giving rise to a homogeneous background for clusters.

**Figure 13:**
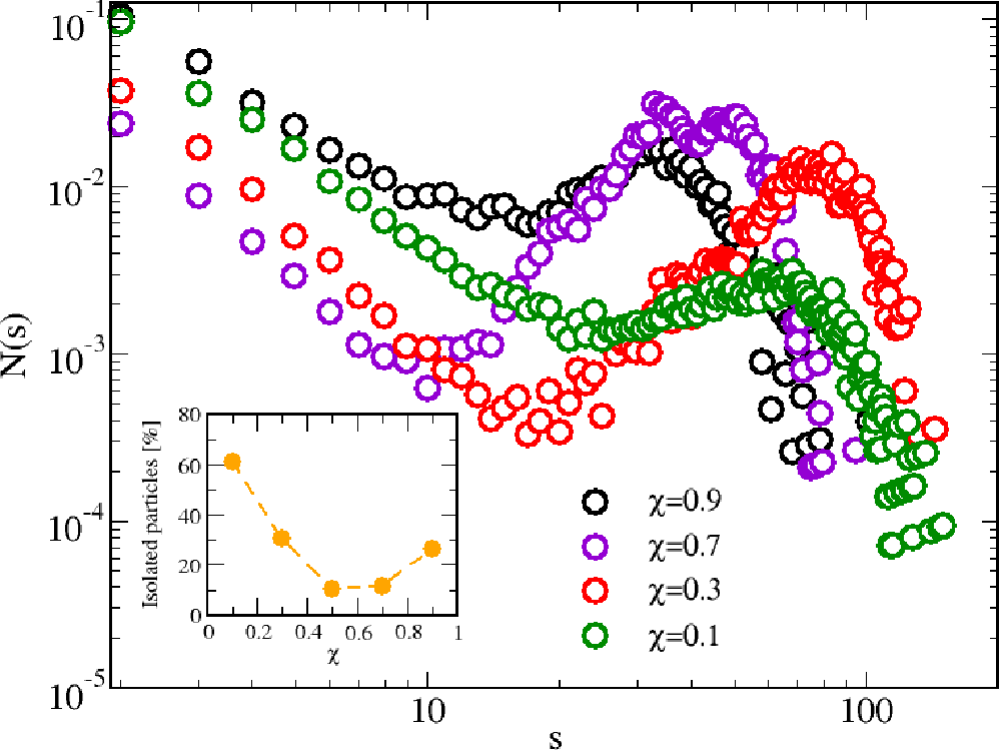
CSD for *T* = 0.25, ε_12_ = 0.75, and a few selected values of χ (in the legend). Inset: fraction of isolated particles vs χ.

Our conclusions, based so far on the sole structural evidence, are confirmed by the microscopic analysis of MC configurations. In Figure 13 we show the CSD for various concentrations: for all concentrations examined, the CSD shows the typical features of a clustered state—specifically, a nonmonotonic decay accompanied by a well-definite local peak at the most probable size. All distributions vanish beyond *s* ≈ 100–200, therefore suggesting the absence of any tendency to percolation. Moreover, we see that comparatively larger clusters are stabilized in the range χ = 0.1–0.5, a feature already noted when analyzing structural correlations. Under the same conditions, the fraction of isolated (mainly HS) particles keeps relatively high, as shown in the inset.

**Figure 14:**
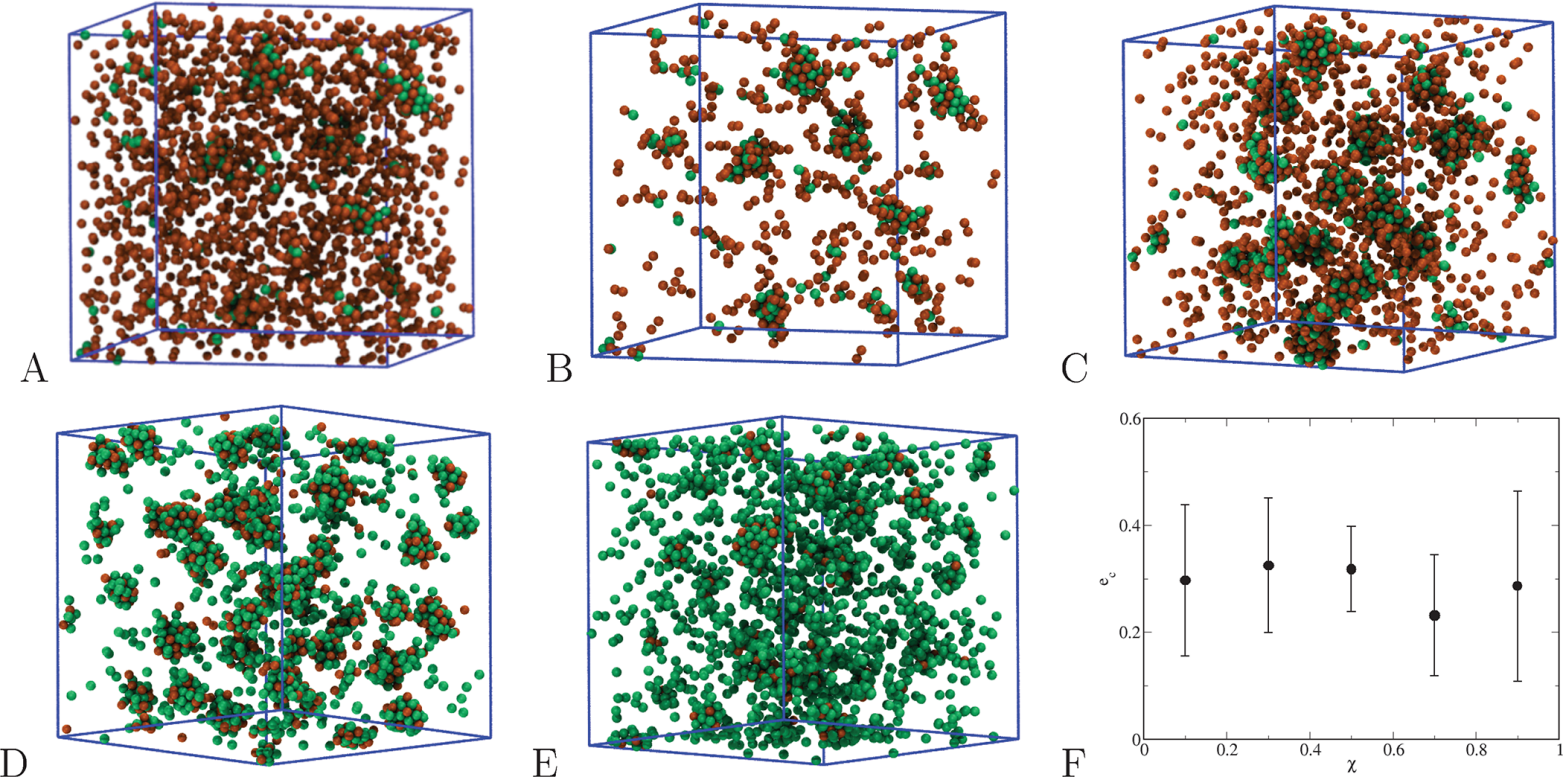
Microscopic configurations for *T* = 0.25 and ε_12_ = 0.75. (A and B) χ = 0.1, with and without isolated particles; (C) χ = 0.3; (D) χ = 0.7; (E) χ = 0.9; (F) average eccentricity with standard deviation of the largest cluster as a function of χ.

The simultaneous occurrence of large clusters and a high number of isolated HS for χ = 0.1 and 0.3 can be better appreciated in the snapshots reported in Figure 14; in particular, a glance at panels A and B—where the same χ = 0.1 configuration is shown with and without isolated particles—indicates that HS (in orange) are homogeneously distributed across the sample. A few large clusters (formed by particles of both species in equal proportions) are immersed in the bath of isolated HS, thus corroborating the picture emerging from the structure. As the HSTY concentration increases (χ = 0.3, panel C) the coupling between HSTY and HS becomes more effective, with an ensuing smaller number of isolated HS. As said before, the optimal condition for mutual aggregation is at equimolarity where practically all HS particles are involved in the formation of aggregates (see Figure 9B and Figure 13, inset). Starting from χ = 0.7 onward (panels D and E), the opposite trend sets in, with a progressively higher number of isolated HSTY particles and the presence of many smaller clusters. The ultimate fate of this process is already known (see Figure 3A): as the HS are completely removed, clusters break apart, and the now pure HSTY fluid is nearly homogeneous.

To gain more information on the cluster structure, we have determined the shape distribution of the largest cluster across configurations, as a function of χ. To this aim, we have followed the method devised in ref (62), in which the cluster eccentricity *e*_c_ is defined as
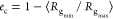
14where *R*_*g*_min__ and *R*_*g*_max__ are, respectively, the minimum and maximum values of the gyration radius *R*_g_ of the largest cluster and the average is taken over sufficiently many configurations. By definition, the value of *e*_c_ ranges from 0 (perfect sphere) to 1 (ellipsoid). The method slightly differs from a similar protocol proposed in ref (63). The behavior of *e*_c_ as a function of χ is reported in Figure 14F, along with standard deviations: clearly, no sensible dependence of *e*_c_ on χ is found, with all values of eccentricity ranging between 0.23 and 0.32. This suggests that the largest clusters have a nearly spherical shape regardless of the relative concentration of the species.

One further remark is worth making in this context. We see in Figure 14D, corresponding to χ = 0.7, that HS are gathered in droplets essentially held together by an outer shell of SALR particles. This surprising outcome indicates the possibility of encapsulating inert particles by a second species with affinity to them, such that they are held apart from the solvent (here only implicitly defined).

**Figure 15:**
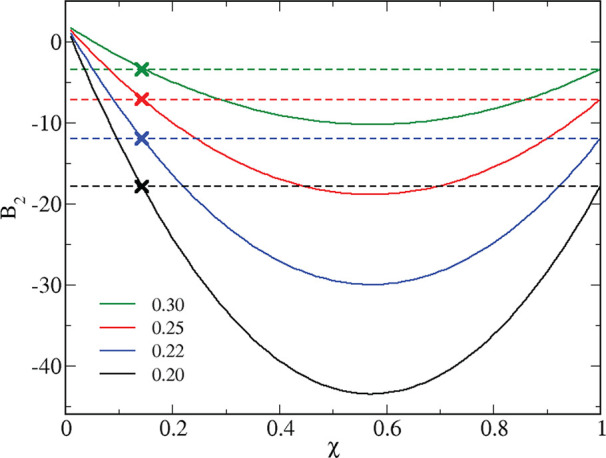
Second virial coefficient of the reference attractive fluids for the mixture with ε_12_ = 0.75 (full lines) and the pure HSTY (dashed lines) vs χ for different temperatures, in the legends. Intersections are marked by crosses.

We end this section by an analysis of *B*_2_ along the same lines followed before, now in terms of HSTY concentration for ε_12_ = 0.75 and fixed temperature. Results are shown in Figure 15: as seen, the two *B*_2_ lines cross for χ = 1 (which is trivial) and for
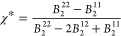
15For the temperatures considered in the figure, we have the pairs [*T*, χ*] = [0.20, 0.1434], [0.22, 0.1432], [0.25, 0.1447], and [0.30, 0.1490], each marked by a cross. Hence, over the rather extended concentration range χ ≈ 0.14–1, the reference attractive mixture is more attractive than the reference attractive HSTY fluid. Accordingly, clusters would appear more structured in the mixture than in the HSTY fluid. At the same time, we must note a discrepancy between the *B*_2_ threshold (χ = 0.14) and the simulation threshold (χ ≈ 0.05). Despite this mismatch, our expectation that clusters are strengthened by replacing even a large fraction of SALR particles with HS is in accordance with the simulation.

Compared to the ε_12_^*^ dependence on *T*, now a weaker dependence of χ* on *T* is found. This is due to a minimum attained by χ* for *T* = 0.22; as a result, χ* slowly changes in the range from *T* = 0.20 to *T* = 0.30, which is a close neighborhood of the minimum. We finally observe that *B*_2_ of the reference attractive mixture reaches a minimum at χ ≈ 0.57. Coherently with our previous conclusion, the optimal conditions for maximizing the number of bonds (hence, for minimizing the energy) are met near equimolarity.

## VI Conclusions

We have carried out Monte Carlo simulations of a model mixture of hard-sphere two-Yukawa particles and hard spheres, being coupled through an attractive square-well potential.

We have ascertained that the inclusion of HS particles can induce the formation of clusters under conditions for which the pure HSTY fluid would not allow for their appearance. As already recognized in ref (54), the tendency of HSTY particles to form clusters can indeed be enhanced by a second species having sufficient affinity to them. We have also verified that the same mechanism can be employed the other way around, so that clustering is hampered in the mixture for the same temperatures where they are present in the pure HSTY fluid.

By simulating the equimolar HSTY–HS mixture at sufficiently low temperatures, it emerges that clusters contain HSTY and HS particles mixed together in equal proportions. Upon moving away from equimolarity, we observe a net preference of the majority species at the cluster surface. In particular, hard spheres can be loosely encapsulated by HSTY particles and, in this way, separated from the (implicit) solvent.

For fixed strength of cross interaction, clustering turns out to be only weakly dependent on the HSTY concentration. In this regard, one of the most interesting results is that clusters can even be induced in a HS fluid by replacing a small fraction of spheres (as low as 5%) with HSTY particles, provided that a sufficiently large attraction (ε_12_ ≥ 0.75) exists between the species. Hence, given a sample composed by inert colloidal particles, clustering can be induced by even marginal chemical substitution with a species with competing interactions and a suitable degree of affinity to the host particles. The optimal condition for aggregation is realized near 50% concentration, where almost all particles participate in the formation of aggregates.

We have placed our results in the more general framework provided in refs (18 and 19), according to which cluster formation takes place under the same conditions where the reference system (without long-range repulsion) would give rise to liquid–vapor phase separation. To substantiate this idea, we have computed the second virial coefficient *B*_2_ of the reference attractive mixture and compared it with the *B*_2_ of the similarly modified HSTY fluid. It emerges that clustering in the mixture—for fixed temperature and density—is invariably obtained as it becomes more attractive than the pure HSTY fluid. Conversely, when the *B*_2_ of the reference attractive mixture falls below that of the pure fluid, clustering is undermined in the HSTY–HS mixture. This indication is in perfect agreement with the evidence got from the structural and the microscopic analysis.

In the near future, we plan to explore the space of model parameters further, in particular by considering other values of the square-well width and also varying the relative size of the two species. To this aim, aside from simulations, we plan to resort on refined, thermodynamically self-consistent integral equation theories of the fluid phase.
